# The preoperative alkaline phosphatase-to-platelet ratio index is an independent prognostic factor for hepatocellular carcinoma after hepatic resection

**DOI:** 10.1097/MD.0000000000005734

**Published:** 2016-12-23

**Authors:** Ya-Qun Yu, Jun Li, Yan Liao, Qian Chen, Wei-Jia Liao, Jian Huang

**Affiliations:** a Department of Hepatobiliary and Pancreatic Surgery; b Laboratory of Hepatobiliary and Pancreatic Surgery, Affiliated Hospital of Guilin Medical University; c Disease Prevention and Control Center of Guilin, Guilin, Guangxi; d Shanghai Center for Systems Biomedicine, Shanghai Jiao Tong University; e Key Laboratory of Systems Biomedicine (Ministry of Education) and Collaborative Innovation Center of Systems Biomedicine, Shanghai, China.

**Keywords:** alkaline phosphatase-to-platelet ratio index, hepatocellular carcinoma, prognosis, survival

## Abstract

A simple, inexpensive, and readily available prognostic index is highly needed to accurately predict the prognosis of hepatocellular carcinoma (HCC). This study aimed to develop a simple prognostic index using routine laboratory tests, alkaline phosphatase-to-platelet count ratio index (APPRI), to predict the likelihood of postoperative survival in HCC patients.

A total of 246 patients with HCC undergoing curative resection were retrospectively analyzed. Cutoff point for APPRI was calculated using receiver operating characteristic curve analysis, and then the patients were divided into the low-APPRI group (APPRI ≤ 4.0) and the high-APPRI group (APPRI > 4.0). The influences of APPRI on disease-free survival (DFS) and overall survival (OS) were tested by the Kaplan–Meier method, and multivariate analysis using Cox regression. Elevated APPRI was associated with age, cirrhosis, and aspartate aminotransferase (AST) in HCC. Univariate analysis showed that APPRI > 4.0, tumor size >6 cm, multiple tumors, Barcelona-clinic liver cancer stages B to C, and AST > 40 U/L were significant predictors of worse DFS and OS. A multivariate analysis suggested that APPRI > 4.0 was an independent factor for DFS (hazard ratio [HR] = 1.689; 95% confidence interval [CI], 1.139–2.505; *P* = 0.009) and OS (HR = 1.664; 95% CI, 1.123–2.466; *P* = 0.011). Preoperative APPRI > 4.0 was a powerful prognostic predictor of adverse DFS and OS in HCC after surgery. The APPRI may be a promising prognostic marker for HCC after surgical resection.

## 1 Introduction

Hepatocellular carcinoma (HCC) is one of the most common cancers, posing severe threats on the health and life of people worldwide. Hepatic resection surgery is an important curative treatment and gold standard for HCC to date. However, the clinical efficacy (prognosis) is far from satisfactory because of the frequent recurrence and poor long-term survival after surgery. Tumor recurrence rates occur in more than 70% of cases at 5 years.^[1,2]^ Therefore, developing new predictive biomarkers to evaluate the postoperative recurrence risk and poor prognosis can provide a new way for early prevention and will be significantly beneficial to patients with cancer considering timely postoperative therapeutic interventions.

The prediction of prognosis plays a key role in effective clinical therapeutic options for HCC patients. Existing prognostic factors such as serum alpha-fetoprotein (AFP), multinodular tumors,^[3]^ tumor Barcelona-clinic liver cancer (BCLC) stage,^[3]^ and tumor size have been identified in previous studies, their prognostic value warrants more investigation and broader applications. However, they have limitations to some extent in sensitivity and specificity. Recently, accumulating evidence has indicated that certain tangible components (e.g., white blood cells [WBCs] and platelets) of the peripheral blood are predictors of prognosis in cancer patients. For instance, a high monocyte count is an independent factor of poor prognosis for patients with HCC^[4]^ and colorectal liver metastasis.^[5]^ Although once primarily recognized for their roles in thrombosis and hemostasis, platelets have been increasingly recognized as a multipurpose cell type. Platelets sequester solubilized tumor-associated proteins^[6]^ and have emerged as central players in the systemic and local responses to tumor growth.^[7,8]^ More importantly, a recent study showed that the serum liver enzyme (aspartate aminotransferase [AST], alanine aminotransferase [ALT], alkaline phosphatase [ALP], and γ-glutamyl transpeptidase [γ-GT]) to peripheral blood tangible component ratio is associated with prognosis in HCC patients^[9–12]^—for example, the preoperative AST-to-platelet ratio,^[9]^ platelet-to-lymphocyte ratio,^[10]^ AST-to-lymphocyte ratio,^[11]^ and γ-GT-to-platelet ratio.^[12]^ However, in patients with HCC, the ALP-to-platelet ratio and indices of based on it as prognostic factors have not yet been established, and the relationship between the clinicopathologic features of HCC, at present, is unknown. Therefore, finding a noninvasive new marker is of great importance to patients with HCC.

The present study aimed to investigate the optimal value of the ALP-to-platelet ratio index (APPRI) and evaluate the correlation of the preoperative APPRI with clinicopathologic features and prognosis in patients with HCC who underwent curative resection. We conducted a retrospective study of 246 patients with HCC to explore their prognostic value for overall survival (OS). The optimal cutoff points for the APPRI were determined. The association between the levels of the APPRI and clinicopathologic characteristics was analyzed. Their prognostic value was explored by univariate and multivariate analysis, and as a basis, initial construction of a preoperative prognostic scoring model.

## 2 Materials and methods

### 2.1 Study population

Source of Specimens and Clinical Data from 256 cases of patients with HCC underwent hepatic resection at the Affiliated Hospital of Guilin Medical University (Guilin, People's Republic of China) from August 1999 to February 2008, and these patients were recruited for this study. These subjects were confirmed by clinical, serological, and ultrasonography (US), computed tomography, magnetic resonance imaging, and pathologic examination. In addition, HCC diagnoses in this study followed the Primary Liver Cancer Clinical Diagnosis and Staging Criteria (Ministry of Health, Beijing, China). The baseline and clinical data include age, gender, liver enzymes (such as AST, ALT, ALP, and γ-GT), serum AFP, hepatitis B virus (HBV) infection, the size and number of tumors, combined liver cirrhosis, and BCLC stage. All of the subjects gave written informed consent, and the local ethics committee approved that this study conformed to the standards of the Declaration of Helsinki. This study was conducted as a retrospective analysis of a prospectively collected computerized database at a single hospital. Among them, 246 patients who met the inclusion criteria were enrolled in this study. These patients met all the following criteria: had been diagnosed with only HCC without portal vein tumor thrombus, lymph node metastasis, or extrahepatic distant metastases; had their clinical background data confirmed; had no lymphatic system disease or other infectious disease, for example, human immunodeficiency virus or hepatitis C virus infection; and were alive during the perioperative period. Our research group continuously monitored these 246 patients by long-term follow-up after surgery, including using the serum AFP test and US examination every 2 months and chest radiography every 6 months during the first 2 postoperative years and at 3- to 6-month intervals thereafter. Computed tomography or magnetic resonance imaging was performed if recurrence was suspected due to an abnormal AFP test or US examination. The mean postoperative follow-up time was 36.7 months (range, 2.0–84.0 months). Disease-free survival (DFS) was measured from the date of surgery to the date of recurrence, metastasis, death, or last follow-up. OS was measured from the date of surgery to the date of death or last follow-up.

### 2.2 Selection of cutoff score

To determine the optimal cutoff value of the APPRI, ALP, and platelets to predict HCC prognosis after liver resection, we analyzed the outcome of 246 HCC patients who underwent liver resection. Receiver operating characteristic (ROC) curve analysis was applied to define the cutoff value of the APPRI (≤4.0 vs >4.0). Other clinicopathologic parameters used were dichotomized: age (≤50 vs >50 years), gender (female vs male), family history (no vs yes), hepatitis B surface antigen (HBsAg) (negative vs positive), AFP level (≤20 vs >20 ng/mL), tumor size (≤6 vs >6 cm), cirrhosis (no vs yes), tumor number (single vs multiple), drinking (no vs yes), BCLC stage (0–A vs B–C), AST (≤40 vs >40 U/L), and recurrence (no vs yes). Subsequently, the clinicopathologic and prognostic significance of the APPRI level in HCC was investigated.

### 2.3 Statistical analysis

SPSS version 13.0 software (SPSS Inc; Chicago, IL) and MedCalc statistical version 11.3.0.0 software (MedCalc Software; Broekstraat 52, Mariakerke, Belgium) were used to analyze the data. Pearson χ^2^ test was used to compare qualitative variables. Univariate analysis was performed to determine the significance of variables using the logistic regression model for the response rate and the Cox regression model for DFS and OS. Survival curves were estimated by Kaplan–Meier analysis, and the log-rank test was used to examine the difference in the survival distributions between groups. Subsequently, the variables with *P* < 0.05 were subjected to multivariate analysis. The Cox proportional hazards regression model was used to determine the independent prognostic factors. A *P* value less than 0.05 was considered to be statistically significant.

## 3 Results

### 3.1 Basic clinical and biochemical data of the examined patients

The clinical and biochemical data of the examined patients are listed in Table 1, including age, median size, AFP, WBC count, lymphocytes, platelets, albumin, globulin, total bilirubin, direct bilirubin, ALT, AST, ALP, γ-GT, and the APPRI. All 246 HCC patients met the inclusion criteria and provided complete clinical background information for our study. Preoperative APPRI was calculated by using the following formula: (ALP value/platelets count) × 10^10^/U.

**Table 1:**
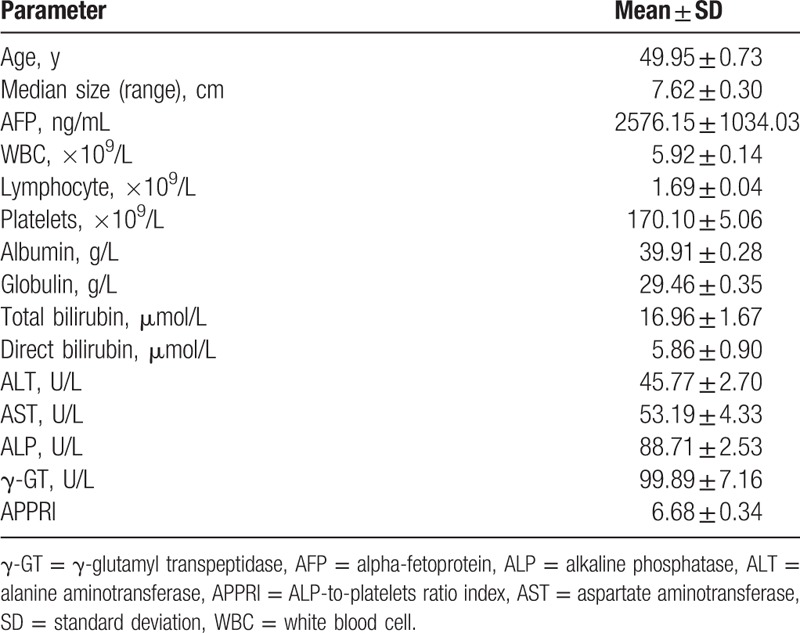
Clinical and biochemical data of examined patients.

### 3.2 An optimal cutoff value for the elevated APPRI

**Figure 1:**
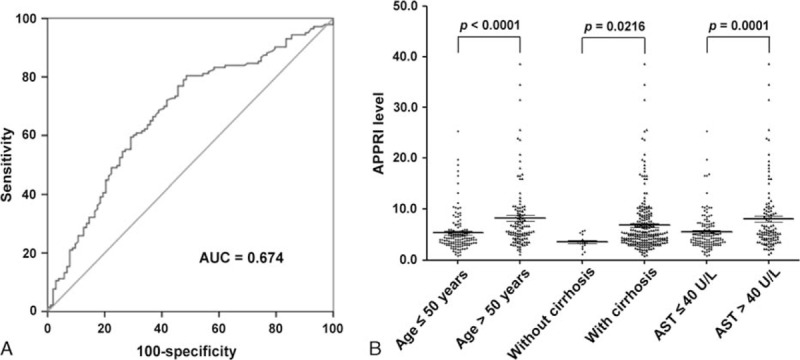
Receiver operating characteristic (ROC) curve and stratified analysis of the preoperative alkaline phosphatase-to-platelet ratio index (APPRI) in hepatocellular carcinoma (HCC) subgroups. (A) ROC analysis was performed to evaluate the prognostic value of the preoperative APPRI. The area under the ROC curve value was 0.674. (B) The sample scatter point distribution of the APPRI levels in different subgroups. All 246 cases of HCC patients were stratified based on age, cirrhosis, and aspartate aminotransferase (AST), thus comparing the preoperative APPRI in different HCC subgroups. The proportions of patients with elevated preoperative APPRI and age >50 years, cirrhosis, and AST > 40 U/L are much higher than those with age ≤50 years, without cirrhosis, and an AST ≤ 40 U/L (*P* < 0.05).

According to the ROC curve, the optimal cutoff value of the preoperative APPRI that had a relatively high specificity was 4.0. The area under the ROC curves was 0.674 with a 95% confidence interval (95% CI) for the area between 0.612 and 0.733. A cutoff value of 4.0 presented a sensitivity of 68.5% and a specificity of 61.2% (Fig. 1A).

### 3.3 Stratified analysis according to age, cirrhosis, and AST

Patients were stratified according to age, cirrhosis, and AST to compare the preoperative APPRI in 2 different HCC subgroups. We found that, when HCC patients were >50 years old, the preoperative APPRI was significantly higher than those aged ≤50 years (8.22 ± 0.60, 5.33 ± 0.33, respectively, t = 4.342, *P* < 0.0001, Fig. 1B). This tendency was also found in HCC patients with cirrhosis in contrast to those without cirrhosis (6.88 ± 0.36, 3.58 ± 0.37, respectively, t = 2.313, *P* = 0.0216, Fig. 1B). In addition, the preoperative APPRI in HCC patients with AST > 40 U/L was increased significantly compared with those with AST ≤ 40 U/L (8.09 ± 0.63, 5.48 ± 0.31, respectively, t = 3.895, *P* = 0.0001, Fig. 1B).

### 3.4 The preoperative APPRI in patients with HCC and its relationship with clinical pathologic characteristics

**Figure 2:**
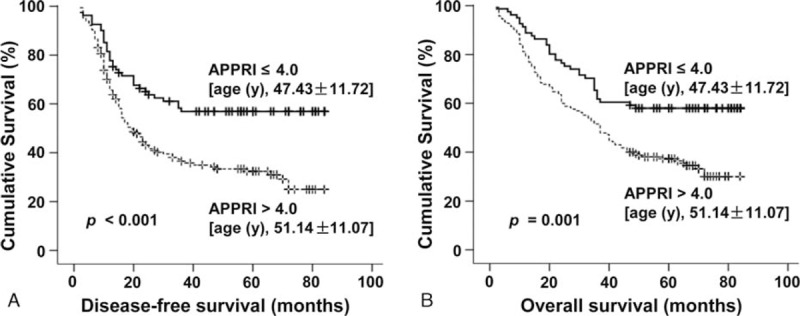
Kaplan–Meier survival analysis of patients with alkaline phosphatase-to-platelet ratio index (APPRI) >4.0 having a shorter disease-free survival (A) and overall survival (B). The mean age in patients with APPRI ≤ 4.0 (n = 81) was 47.43 ± 11.72 years, and 51.14 ± 11.07 years in patients with APPRI > 4.0 (n = 165) (*P* = 0.017). The dashed line represents the APPRI > 4.0 (n = 165), whereas solid line represents the APPRI ≤ 4.0 (n = 81).

As shown in Table 2, the relationship between the preoperative peripheral blood APPRI and clinical pathologic characteristics was investigated. One hundred sixty-five patients (67.07%) identified as the high-APPRI group had an elevated APPRI (>4.0), and 81 patients (32.93%) were identified as the low-APPRI (≤4.0) group. The preoperative APPRI level was closely correlated with age (>50 years, χ^2^ = 8.730; *P* = 0.003), combination of liver cirrhosis (χ^2^ = 5.112; *P* = 0.024), and the serum AST level (χ^2^ = 12.928; *P* < 0.001). The mean age (51.14 ± 11.07) in patients with APPRI > 4.0 was higher than that (47.43 ± 11.72) in APPRI ≤ 4.0 group (*P* = 0.017) (Fig. 2). No obvious correlations with gender, tumor family history, HBsAg, median size, number of tumors, drinking, BCLC stage, recurrence, or serum AFP level were observed (all *P* > 0.05). The data demonstrated that the preoperative APPRI has a strong correlation with older age, cirrhosis, and high AST level, suggesting that these factors may be related to patients with HCC in liver damage.

**Table 2:**
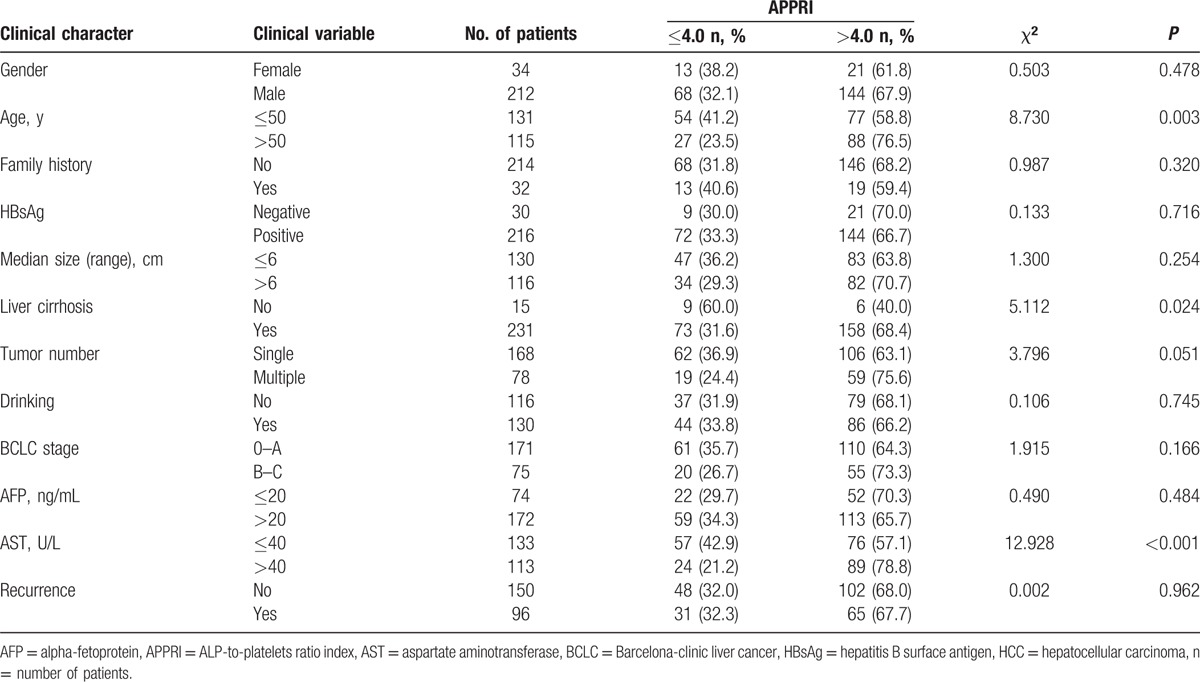
Correlation between the clinicopathologic variables and APPRI level in HCC.

### 3.5 Association of the APPRI or clinical pathologic index between postoperative DFS and OS

Kaplan–Meier survival analysis showed that an APPRI > 4.0 was associated with a shorter DFS (Fig. 2A) and OS (Fig. 2B). Univariate analysis revealed that an obvious association existed between clinical parameters and both DFS (Table 3) and OS (Table 4). The mean DFS in patients with APPRI ≤ 4.0 was 54.50 months (95% CI, 46.90–62.11) compared with 36.87 months (95% CI, 31.77–41.97) in patients with APPRI > 4.0 (*P* < 0.001). The mean OS rates in the APPRI ≤ 4.0 group and APPRI > 4.0 group were 58.60 months (51.88–65.31) and 44.05 months (39.30–48.80), respectively (*P* = 0.001). In addition to the high-APPRI group (APPRI > 4.0), a size of the tumor >6 cm, multiple tumors, a BCLC stages B to C, and a serum AST level >40 U/L were also associated with a shorter DFS and OS (*P* < 0.001); recurrence was also associated with a shorter OS (*P* = 0.028, Table 4).

**Table 3:**
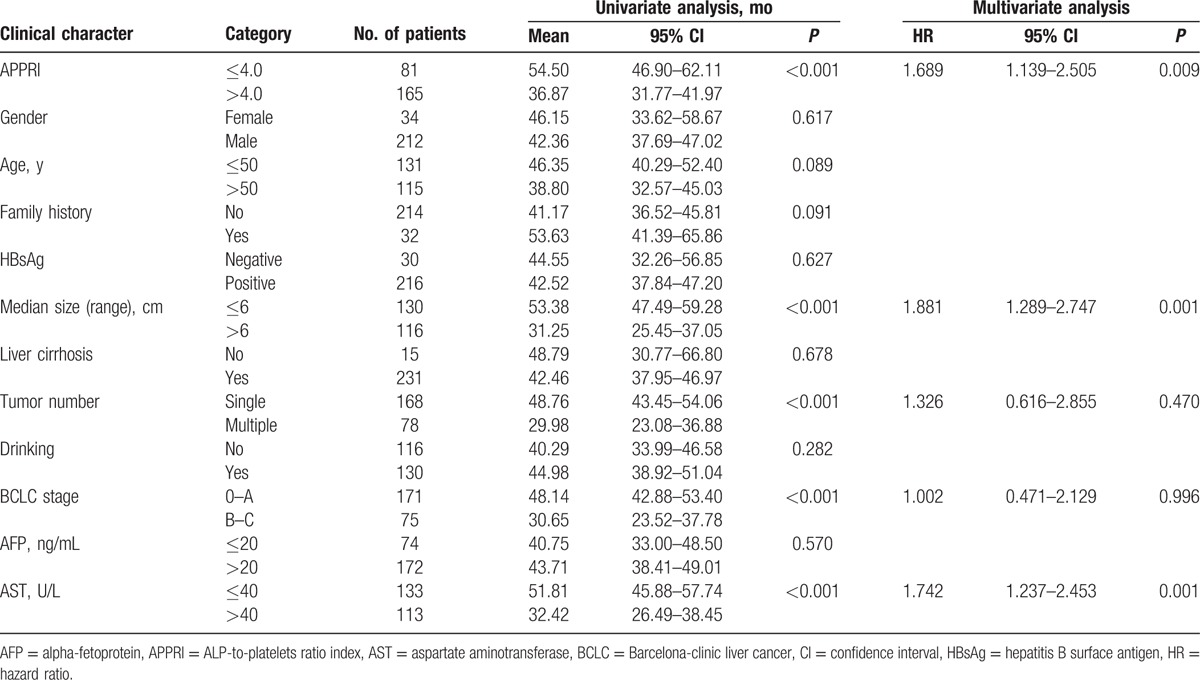
Association between APPRI level or clinical parameters and DFS.

**Table 4:**
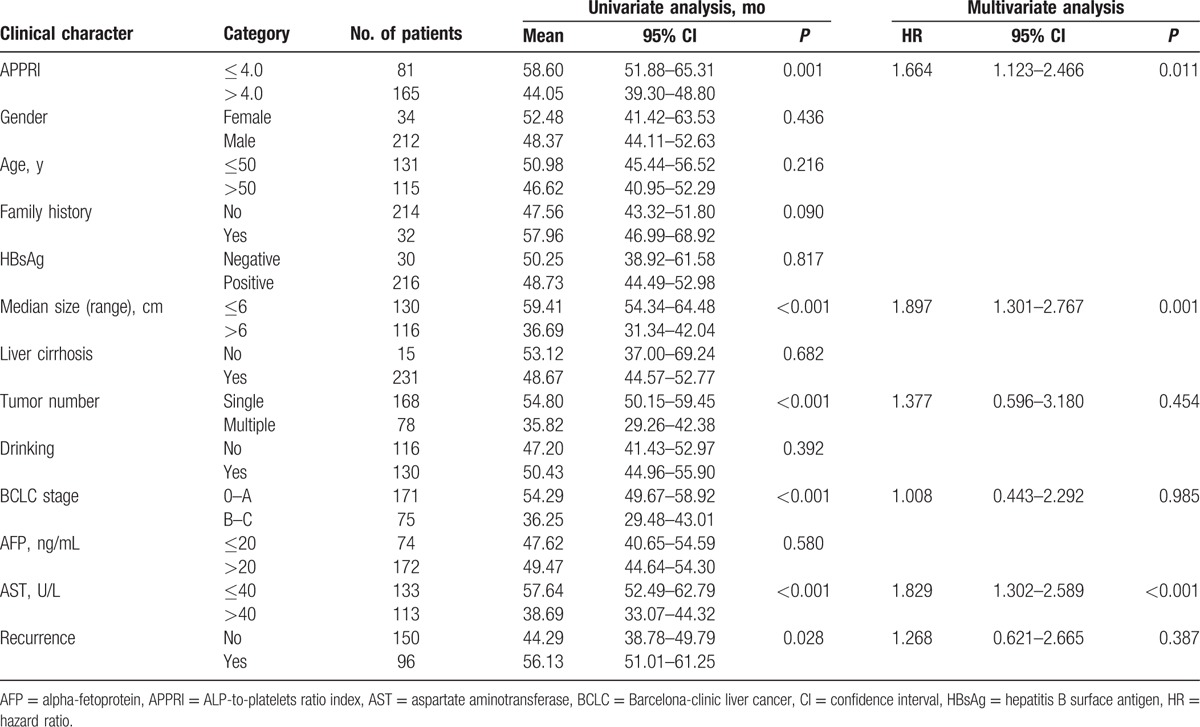
Association between APPRI level or clinical parameters and OS.

### 3.6 Independent predictors of DFS and OS in the stepwise multivariate Cox proportional hazards model

The Cox proportional hazards model was used to examine the association between clinicopathologic factors and DFS/OS after the surgical resection of HCC. After adjusting for other confounding factors, except for recurrence associated with OS, 5 associated factors (high APPRI, size of tumor >6 cm, multiple tumors, BCLC stages B to C, and AST > 40 U/L) were analyzed for DFS and OS using the stepwise multivariate Cox proportional hazards model. Three factors were significant in the Cox proportional hazards model. The hazard ratio (HR), 95% CI, and *P* values of the 3 independent predictors are listed in Tables 3 and 4. The stepwise multivariate Cox proportional hazards model revealed that a high APPRI (HR, 1.689; 95% CI, 1.139–2.505; *P* = 0.009), a size of tumor >6 cm (HR, 1.881; 95% CI, 1.289–2.747; *P* = 0.001), and a serum AST level >40 U/L (HR, 1.742; 95% CI, 1.237–2.453; *P* = 0.001) were independent predictors of DFS (Table 3). A high APPRI (HR, 1.664; 95% CI, 1.123–2.466; *P* = 0.011), a size of tumor >6 cm (HR, 1.897; 95% CI, 1.301–2.767; *P* = 0.001), and an AST > 40 U/L (HR, 1.829; 95% CI, 1.302–2.589; *P* < 0.001) were independent predictors of OS (Table 4).

### 3.7 Kaplan–Meier analysis of DFS and OS in 246 HCC patients based on statistically significant clinical parameters

**Figure 3:**
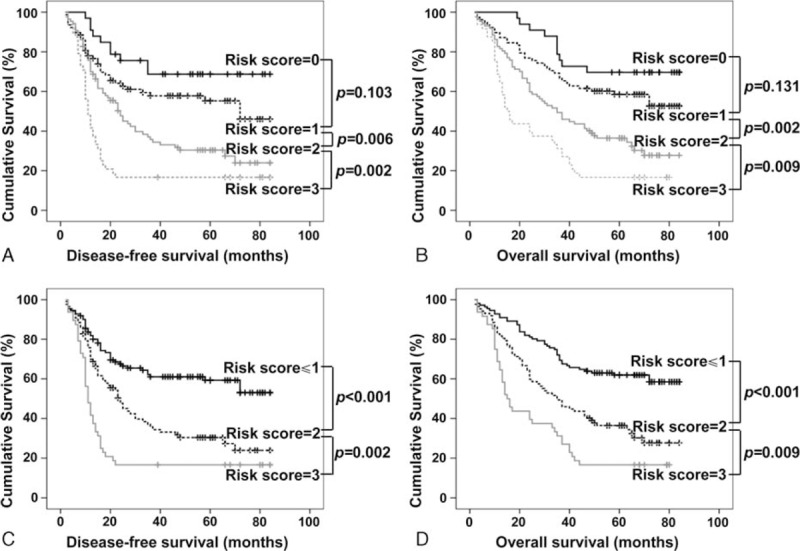
The disease-free survival (DFS) and overall survival (OS) for patients with various risk scores (RSs) according to the independent predictors. (A and B) The Kaplan–Meier curves for the 5 groups of patients showed that there were no significant differences in the DFS (A) and OS (B) rates of patients with scores of 0 to 1 (all *P* > 0.05). However, there were significant differences in the DFS (A) and OS (B) of patients with scores from 1 to 4 (all *P* < 0.01). (C and D) There were also significant differences in the DFS (C) and OS (D) after patients with a RS = 0 or 1 were merged (all *P* < 0.01).

We established a preoperative prognostic score model by calculating the number of independent predictors (APPRI, size of tumor, and AST) for each patient. Each positive factor as a score of 1, and then the patients were divided into 4 categories according to their risk scores (RSs) (0–3). For example, a “risk score = 0” indicates patients without any of the above factors, and this group accounted for 13.41% (33/246) of the patients, and a “risk score = 3” indicates patients with all 3 factors, and this group accounted for 19.51% (48 of 246) of the patients carrying all 3 factors (Fig. 3). Because no significant difference was observed in DFS and OS between patients whose RS was 0 or 1 (Fig. 3A and B; *P* = 0.103 and 0.131, respectively), these patients were merged as the score ≤1 group. By combining 3 independent predictors, patients with different RSs showed distinguishable DFS (RS ≤ 1 vs RS = 2, *P* < 0.001; RS = 2 vs RS = 3, *P* = 0.002, Fig. 3C) and OS (RS ≤ 1 vs RS = 2, *P* < 0.001; RS = 2 vs RS = 3, *P* = 0.009, Fig. 3D). Surprisingly, the proportion of HCC patients with RS = 3 was very high, occupying 19.51% (48 of 246) of all patients. The DFS and OS in 48 patients with a score of 3 decreased sharply, and all of these patients showed a much shorter DFS and OS.

## 4 Discussion

ALP is an enzyme widely distributed in the human tissues of the liver, bone, intestine, and kidney, and low-density ALP is measured in normal human serum. However, serum ALP, mainly from the liver, has a high specificity. Except for ALP, serum liver enzymes such as ALT and AST are routinely tested in HCC patients. These enzymes are commonly elevated in patients with HCC and thus, may reflect the status of liver injury.^[13]^ Of the liver enzymes, ALT, AST, ALP, and γ-GT have long been recognized to play potential roles in the diagnosis of HCC.

Our present study demonstrated that the preoperative APPRI, an index calculated from 2 conventional available laboratory tests, might be a potential predictive marker for patients with HCC. According to the ROC curves, the cutoff value of 4.0 appeared to be the most suitable reference value for the preoperative APPRI with a sensitivity of 60.3% and a specificity of 67.2% for predicting HCC. We also found that high levels of the APPRI showed a positive correlation with age (>50 years), cirrhosis, and AST (>40 U/L). It is speculated that the progress of cirrhosis was promoted by a high APPRI level. Currently, the process of liver fibrosis, cirrhosis, and development to HCC have achieved some consensus, and it is clear that chronic HBV infection is the most important factor in HCC development.^[14–16]^ In China, the vast majority (about 95%) of HCC patients have HBV infection and liver cirrhosis, and patients with liver cirrhosis, portal hypertension, hypersplenism, and low platelet and WBC levels develop HCC; on the other hand, due to liver damage, serum liver biochemistry enzyme indexes are increased—for example, ALP, AST, ALT, and γ-GT levels—primarily leading to an increased APPRI. Furthermore, platelets have been well established to play a key role in liver fibrosis,^[17,18]^ cirrhosis,^[19]^ tumor growth, and metastasis.^[20–23]^ Thrombocytopenia exacerbates liver fibrosis.^[17]^ By contrast, increasing the platelet count suppresses hepatic fibrosis.^[18]^ Other studies have also suggested that small HCCs usually arise in cirrhosis, often associated with thrombocytopenia.^[19]^ We speculate that the level of APPRI may correlate with HCC progress, which would have important clinical application value.

In the present study, the prognostic role of preoperative APPRI in patients with HCC was investigated. The data from 246 patients with HCC who underwent liver resection were analyzed. Our results revealed that a preoperative APPRI > 4.0 was an independent predictor of DFS and OS in HCC patients after hepatectomy. To our knowledge, this study is the first article in investigating the prognostic significance of the preoperative APPRI in HCC patients.

Based on the results of univariate analysis, except for APPRI, we found that a tumor size >6 cm, multiple tumors, BCLC stages B to C, and AST were associated with a short DFS and OS. The prognostic relevance of the tumor size,^[24,25]^ tumor number,^[26]^ BCLC stage,^[3]^ and AST^[27,28]^ for survival in HCC patients was confirmed by previous studies. In Roayaie et al's^[25]^ report, tumor size was an important determinant for the survival of HCC patients. Obviously, individuals with multinodular HCC tumors had a relatively short survival and poor prognosis compared with those with a single tumor.^[26]^

The findings in the multivariate analysis showed that a preoperative APPRI > 4.0, a tumor size >6 cm, and an AST > 40 U/L were independent prognostic markers of DFS and OS in HCC patients. This finding is consistent with previously reported results that tumor size may act as an independent prognostic factor for resected small HCC.^[29]^ Generally, small HCC tumors have a better prognosis.^[25]^

From our study, the optimal cutoff value of preoperative APPRI for predicting the prognosis of HCC was 4.0. Calculating the preoperative APPRI is a simple method for the judgment of prognosis in HCC patients. However, our survey also has some limitations. Many other factors affecting APPRI, such as antiviral therapy before surgery, may impair the accuracy of the prognostic prediction. In the follow-up, we found that the success of the antiviral therapies might decrease HCC recurrence and improve postoperative survival in a minority of patients who receive antiviral therapies. Yet, we discovered that only about 5% of the HCC patients who were conventional candidates for antiviral treatment receive the antiviral therapies, while the vast majority of patients give up antiviral treatment because of economic or other reasons. In addition, the AFP level and HBV infection might also affect the prognosis of HCC, although the effect was not significant in our study, probably due to the limited number of patients. The prognostic role of the APPRI only by retrospectively analyzing the clinical data of 246 patients from our hospital, it would be necessary to validate the prognostic significance of the APPRI levels in a larger cohort of HCC patients.

We found a significant elevation in the APPRI levels before surgery, which independently predicted prognosis in HCC patients. In addition, this effect was also significantly increased when we combined the tumor size and AST; based on the 3 parameters, we successfully constructed a model of the preoperative prognostic score. This provided a new RS for HCC. However, the small sample size of the present study limits its clinical value. The validation of the prognostic value of the preoperative prognostic scoring model should be best warranted in future clinical trials from other institutions. A prospective study with a larger population should be conducted to justify our studies.

In conclusion, our data suggest that an elevated preoperative APPRI is an independent predictor of poor prognosis for patients with HCC after hepatic resection. HCC patients with an elevated APPRI should be subjected to close follow-up and timely postoperative therapeutic intervention to improve their life quality. The combined use of the existing prognostic biomarkers with the APPRI will offer incremental predictive information and will substantially enhance the sensitivity and specificity of predicting prognosis in HCC patients. As expected, a size of the tumor >6 cm and an AST > 40 U/L were also independent prognostic factors in our study. It is likely that we could use the APPRI to evaluate the prognosis of HCC patients. In addition, the above 2 factors (tumor size and AST) comprehensively will improve the predicting accuracy.

Most importantly, the APPRI, as a novel predictive tool in clinical practice, has major advantages concerning its simplicity and objectivity. It is derived from 2 easily available laboratory results (ALP level and platelet count). Both the ALP level and platelet count are routine tests performed in HCC patients in clinical practice, and no extra tests are needed. Therefore, it is simple, objective, and inexpensive. In addition, the APPRI is expected to provide more biomarkers of diagnosis and monitoring to implement personalized medicine in patients with cancer.
